# Pondering Ponds: Exploring Correlations Between Cloacal Microbiota and Blood Metabolome in Freshwater Turtles

**DOI:** 10.1007/s00248-025-02556-7

**Published:** 2025-05-23

**Authors:** T. Franciscus Scheelings, Saritha Kodikara, David J. Beale, Thi Thu Hao Van, Robert J. Moore, Lee F. Skerratt

**Affiliations:** 1 Melbourne Veterinary School, Faculty of Science, The University of Melbourne, Werribee, VIC 3030 Australia; 2 School of Mathematics and Statistics, Faculty of Science, The University of Melbourne, Parkville, 3052 Australia; 3 Environment, Commonwealth Scientific and Industrial Research Organisation (CSIRO), Ecosciences Precinct, Dutton Park, QLD 4102 Australia; 4 School of Science, RMIT University, Bundoora West Campus, Bundoora, VIC 3083 Australia

**Keywords:** Chelonian, Gut, Metabolome, Microbiota, Physiology, Turtle

## Abstract

The gut microbiota of vertebrates significantly influences host physiology, yet little is known about how habitat factors shape microbiotas in non-human species, especially freshwater turtles. This study explores the relationship between cloacal microbiota and serum metabolome in eastern longneck turtles (*Chelodina longicollis*), marking the first such investigation in chelonians. By comparing microbiotas from two distinct pond environments, we applied a multi-omics approach combining 16S rRNA sequencing and metabolomic profiling. Results showed that location influenced microbial composition and metabolic profiles, with dominant bacterial phyla Pseudomonadota, Actinomycetota, and Bacillota, and distinct families linked to differences in microbial diversity. Notably, turtles from one pond displayed an unusually high proportion of Actinomycetota. We also found a clear connection between microbiota diversity and metabolome, suggesting certain bacterial combinations impact host physiology. These findings offer important insights into the complex interaction between microbial communities and metabolism in freshwater turtles, a highly threatened group. This research emphasises the value of integrating microbiota and metabolomic data in conservation strategies and highlights the need for further longitudinal studies to explore the dynamic host-microbiota relationship in these understudied species.

**Supplementary Information:**

The online version contains supplementary material available at 10.1007/s00248-025-02556-7.

## Introduction

Residing within the gut of vertebrates is an array of microorganisms that influence host phenotype by governing diverse physiological processes including macronutrient digestion and assimilation [1], regulation of mucosal and systemic immune responses [2], maintenance of organ health [3], modulation of endocrine function [4], and manipulation of behaviour [5]. In fact, the host-microbiota relationship is so vital to vertebrate homeostasis that it is considered a key driver of evolution [6], with several studies correlating vertebrate gut microbial diversity to their phylogenetic history [7–10]. These effects on host physiology are mediated by the trillions of genes harboured within the complex community of gastrointestinal microorganisms which facilitate the transformation of carbohydrates, proteins, and lipids into various metabolites that are absorbed by the host and impact homeostasis [11]. Understanding these functional relationships is critical to unravelling how species adapt to environments, especially in an age of unparalleled climactic and environmental upheaval.

While identifying the specific microbes present in hosts and their varying abundances is important, a central goal of microbiome research is to explore the functional significance of microbial communities and their effects on host physiology [12]. This can be difficult to achieve in free-ranging animals, and relatively few studies on wild microbiotas have incorporated functional analyses into their research [13–15]. Instead, most investigations consider host physiological variation on a superficial level such as age [16], sex [17], reproductive status [18], or geographic location [19]. However, recent advances in analytical tools, including various ‘omics’ technologies, now enable researchers to collect data on microbial functions, providing a deeper level of insight into microbiota studies. This shift enables us to move beyond simply identifying what microbes are present to understanding what roles they play and how they influence their host.

Variations in the human plasma metabolome have been closely linked to gut microbiotas with potential implications for host health [20, 21]. For example, the bacterial species *Eubacterium rectale* has been shown to decrease plasma levels of hydrogen sulfite, a toxin that affects cardiovascular function [20]. Additionally, short-chain fatty acids (SCFAs) derived from bacterial consumption of undigestible fibre are known to protect against obesity and diabetes in people [22, 23]. Conversely, increased bacterial production of compounds such as N-nitroso, ammonia, and hydrogen sulfite can induce reactive oxygen species resulting in DNA damage and activation of inflammatory pathways [24]. Furthermore, deoxycholic acid, a secondary bile acid manufactured by the microbiota, promotes the development of hepatocellular carcinoma [25]. Thus, molecules produced by microbial metabolism within the gut can have both a beneficial and harmful effect on the host, but these relationships are poorly understood in non-human species, especially in lesser-studied vertebrates such as reptiles.

Despite the increased awareness of the role that the microbiota plays in animal health and fitness, there is a taxonomic bias in the current breadth of investigations. Animal microbiota studies are dominated by research involving domestic species, and by far the most common group to be investigated are mammals [26]. There are relatively few investigations into the microbial communities of wild freshwater chelonians [27–32], and there have been limited attempts to link microbial community composition to host physiology in these species [33]. Chelonians are one of the most threatened vertebrate groups on the planet [34, 35], and conservation efforts are potentially hampered by a lack of understanding of how their microbial symbionts drive physiology and adaptability. A greater emphasis needs to be placed on decoding the chelonian microbiota and its role in host homeostasis, especially given that in modern conservation there is now consideration to preserving holobionts rather than focusing solely on the host [36]. Understanding how gut microbes affect chelonian physiology is essential, particularly as species face rapid environmental degradation due to climate change and habitat loss. Investigating this relationship in freshwater turtles, a largely overlooked taxa will provide insights into how biodiversity might be preserved in the face of environmental stressors.

Therefore, the aims of this investigation were to explore the cloacal microbial communities of two populations of eastern longneck turtles (*Chelodina longicollis*) occupying separate habitats, and to determine their functional effect on the host metabolome. It is hoped that by integrating multi-omics datasets, including microbial and metabolomic information in this system, we will gain a better understanding of the functional role that the microbiota has in a poorly studied taxon and provide further insight into the co-evolutionary history of the vertebrate-microbiota relationship.

## Materials and Methods

### Study Populations

Turtles were trapped from two waterbodies, Duck Pond (DP) and Ivanhoe Wetland (IW), in the Darebin Parklands, Alphington, Victoria, Australia, in January 2023 (the Austral summer of 2022/23). The Darebin Parklands are located in an urban setting in Melbourne, which is the second largest city in Australia with a population of approximately 4.9 million people. Within this park are multiple freshwater ponds known to be populated with turtles. These study sites were a subset of a larger investigation exploring the effects of geography on freshwater chelonian microbiotas [37], and were chosen based on differences in topography and local factors. DP is an old landfill site that became a permanent waterbody 40 years ago; it has a water surface area of approximately 4000m^2^. It is located in a highly visited public area, with sparse vegetation along the banks and a large population of waterbirds. IW is a manmade wetland constructed during the early 1990s and is closely associated with the Darebin Creek system; during the cooler months, it has a water surface area of 3600m^2^ which can reduce to around 2800m^2^ in warm summers. IW is less accessible to the public, features dense vegetation along the water’s edge, and contains extensive reed and grass beds within the waterbody. DP and IW are separated by approximately 200 m in a straight line.

### Sample Collection

At both sites, turtles were trapped using large, 6 hoop fyke nets with 28-mm black, knotless mesh and a 2.5-m wing with a 48-cm drop and baited with chunks of lamb or chicken liver. Six to ten nets were placed into each waterbody and left overnight. In the morning, traps were brought back to shore and turtles removed for sample collection. We elected to only sample adult turtles as these were the only life stage large enough for complete sample collection. Once turtles had been removed from the nets, they were measured (straight carapace length) and weighed using domestic kitchen scales. They were then placed into dorsal recumbency, and a sterile cotton swab was inserted into the cloaca and twirled so that it contacted the cloacal mucosa. The swab was then retracted, and the tip cut using flame-sterilised wire cutters and stored in 1 ml of ZymoBIOMICS DNA/RNA Shield (Integrated Sciences) in a sterile Eppendorf tube. The tubes were then frozen and stored at − 80 °C until DNA extraction could occur.

Once cloacal swabbing had been completed, blood was collected by placing the turtle into dorsal recumbency and an area of skin over the jugular vein was prepared using alcohol wipes. Blood was obtained from the jugular vein while the turtle’s head was retracted within the shell. A 25G needle attached to a 3-ml syringe was used, and a volume not exceeding 1% of body weight was obtained from each individual. Immediately after collection, blood was transferred into a gel clot activator tube (Sarstedt AG & Co., Nümbrecht, Germany). Blood tubes were then placed into a portable ice pack and taken back to the laboratory for processing. Once back at the laboratory, the tubes were centrifuged at 3000 g for 10 min, and the resultant serum removed and stored at − 80 °C until analysis. All turtles were released alive at their point of capture following sample collection.

### Microbial DNA Extraction

DNA was extracted using the ZymoBIOMICS DNA Miniprep Kit (Integrated Sciences) according to the manufacturer’s instructions. Following extraction, DNA was stored at − 80 °C until amplicon sequencing could take place.

### 16S rRNA Gene Amplicon Sequencing

The V3-V4 region of 16S rRNA genes were PCR amplified with forward primer 5′ ACTCCTACGGGAGGCAGCAG 3′ and reverse primer 5′ GGACTACHVGGGTWTCTAAT 3′ using Q5 high-fidelity polymerase (New England Biolabs) with a dual barcoding strategy [38]. The PCR cycling parameters were 98 °C for 1 min, 35 cycles of 98 °C for 10 s, 49 °C for 30 s, and 72 °C for 30 s, followed by a 10-min extension at 72 °C. Sequencing was performed on an Illumina MiSeq system (2 × 300 bp).

### Data Processing

Sequence data was analysed using Quantitative Insights into Microbial Ecology 2 (QIIME2) version 220.6 [39], using the Divisive Amplicon Denoising Algorithm (DADA2) plugin for quality filtering, denoising, chimaera detection, and amplicon sequence variant (ASV) calling [40]. ASVs were taxonomically classified using the SILVA database (v138.1) [41]. An ASV abundance table with taxonomic assignments was produced for further analysis.

### Functional Omics Analysis

Metabolites and lipids were extracted from 100 μL of turtle serum using a one-pot extraction method previously described [42]. Briefly, serum was quenched with 450 μL of ice-cold (− 20 °C) methanol:ethanol (50% *v*/v; LiChrosolv®, Merck, Darmstadt, Germany), and vortexed for 2 min. The samples were centrifuged (Centrifuge 5430R, Eppendorf, Hamburg, Germany) at 14,000 rcf at 4 °C for 5 min to pellet the protein. The supernatant was transferred and filtered using a positive pressure manifold (Agilent PPM48 Processor, Agilent Technologies, Santa Clara, California, USA) with Captiva EMR cartridges (40 mg, 1 mL; Agilent Technologies, Mulgrave, VIC, Australia) to separate the lipid and metabolite fraction [43, 44]. Two stable-isotope internal standards were used throughout the extraction; 100 ppb of l-phenylalanine (l-^13^C) was spiked first with the EtOH:MeOH, followed by a 200 ppb of succinic acid (1,4-^13^C_2_) added when samples were reconstituted. The internal standards were sourced from Cambridge Isotope Laboratories (Andover, MA, USA). The residual relative standard deviation (RDS%) of the raw internal standards were 15.1% (l-phenylalanine, l-^13^C) and 0.8% (succinic acid, 1,4-^13^C_2_), and were used to normalise the metabolomics data prior to statistical analysis. A pooled biological quality control (PBQC) sample (*n* = 3), consisting of 5μL from each sample, was randomly analysed within the analytical sequence. Additionally, a set of authentic amino acids and organic acids was analysed as a matrix-free QAQC sample (*n* = 3). The PBQC and QAQC results were within 10% RSD.

Central carbon metabolism metabolites were analysed on an Agilent 6470 LC-QqQ-MS coupled with an Agilent Infinity II Flex UHPLC system (Agilent Technologies, Santa Clara, California, USA) using the Agilent Metabolomics dMRM Database Method [45]. Untargeted non-polar lipids were analysed using an Agilent 6546 Liquid Chromatography Time-of-Flight Mass Spectrometer (LC-QToF) with an Agilent Jet Stream source coupled to an Agilent Infinity II UHPLC system (Agilent Technologies, Santa Clara, CA, USA) [45–48]. Lipid annotation was performed using acquired Auto MSMS data on pooled PBQC samples obtained at 20 eV and 35 eV collision energy. Collected Auto MSMS data were then processed using the MassHunter Lipid Annotator tool (Version 1.0, Agilent Technologies, USA), and a sample specific PCDL database was created and used to putatively identify lipids in all analysed samples based on MSMS spectra and library threshold score of 0.8 [49].

### Statistical Analyses

ASV abundance data was analysed in R, utilising the package ‘phyloseq’ [50]. Alpha diversity was explored using Observed ASVs, Inverse Simpson, and Shannon index estimates. Alpha diversity was tested for normality using the Shapiro-Wilks test and for normally distributed data comparisons between sites were made using the Welch two sample *t*-test, and for non-normally distributed data comparisons were made using the Wilcoxon rank sum test. Beta diversity was investigated using principal coordinate analysis (PCoA) on weighted and unweighted UniFrac distances. We used the *adonis2* function from the R package ‘vegan’ to perform PERMANOVAs to compare beta diversity between locations [51]. For abundance, testing read counts were transformed to proportions per sample (i.e. total sum normalisation (TSN)) prior to calculating the distances and dissimilarities [52].

In order to investigate the relationships between microbiota composition and omic profiles, we performed a multi-omics analysis across the entire dataset using the R package ‘mixOmics’ [53]. For this analysis, we divided our omics data into metabolomics (amino acids, nucleotides, organic acids, alcohols, vitamins, and carbohydrates) and lipidomics. Due to the long chemical structure names assigned to lipids, we renamed these compounds using a simple numbering system, and the original names can be found in the supplementary material (Table S1). First, we performed cumulative sum scaling (CSS) normalisation on the microbiota data to correct for any bias in the assessment of differential abundance that may have been introduced by TSN. We then filtered all data (microbiota, metabolomics, and lipidomics) by removing low counts of < 0.01% and we chose to assess microbiota data at a Family level due to high sparsity at ASV level. We integrated the omic datasets using DIABLO (Data Integration Analysis for Biomarker discovery using Latent variable approaches for Omics studies), a supervised multivariate approach that maximises shared information across multiple omics datasets while modelling their relationship with a categorical variable [53]. DIABLO applies a sparse selection strategy to identify the optimal number of variables from each dataset, enhancing discriminative power. DIABLO also facilitates the exploration of correlations within and between selected features across datasets. We examined these associations in the first two components. To visualise the relationships among selected features, we generated a circos plot, where different feature types are arranged in a circular layout, with links representing strong positive or negative correlations (*r* > 0.5). Additionally, we created a clustered image map (CIM) to further explore the multi-omics molecular signature expression across samples. Finally, we evaluated model performance using tenfold cross-validation repeated 10 times. Classification error rates were weighted based on the correlation between predicted co as well as weighting the classification error rate from each dataset according to the correlation between the predicted components and the Y outcome. For all statistical analyses, significance was accepted if *p* < 0.05.

To determine the origin of metabolites (host, microbe, environment, or co-metabolism), we used the online resource MetOrigin [54]. We did this for all explanatory metabolites for components 1 and 2 as predicted by our model. As the eastern longneck turtle (or any other Australian species of freshwater turtle) does not have a metabolic pathway described in the KEGG database (with MetOrigin queries), we used a nearest match species, the red-eared slider (*Trachemys scripta elegans*), a freshwater turtle from North America.

## Results

### Microbiota Results

**Fig. 1:**
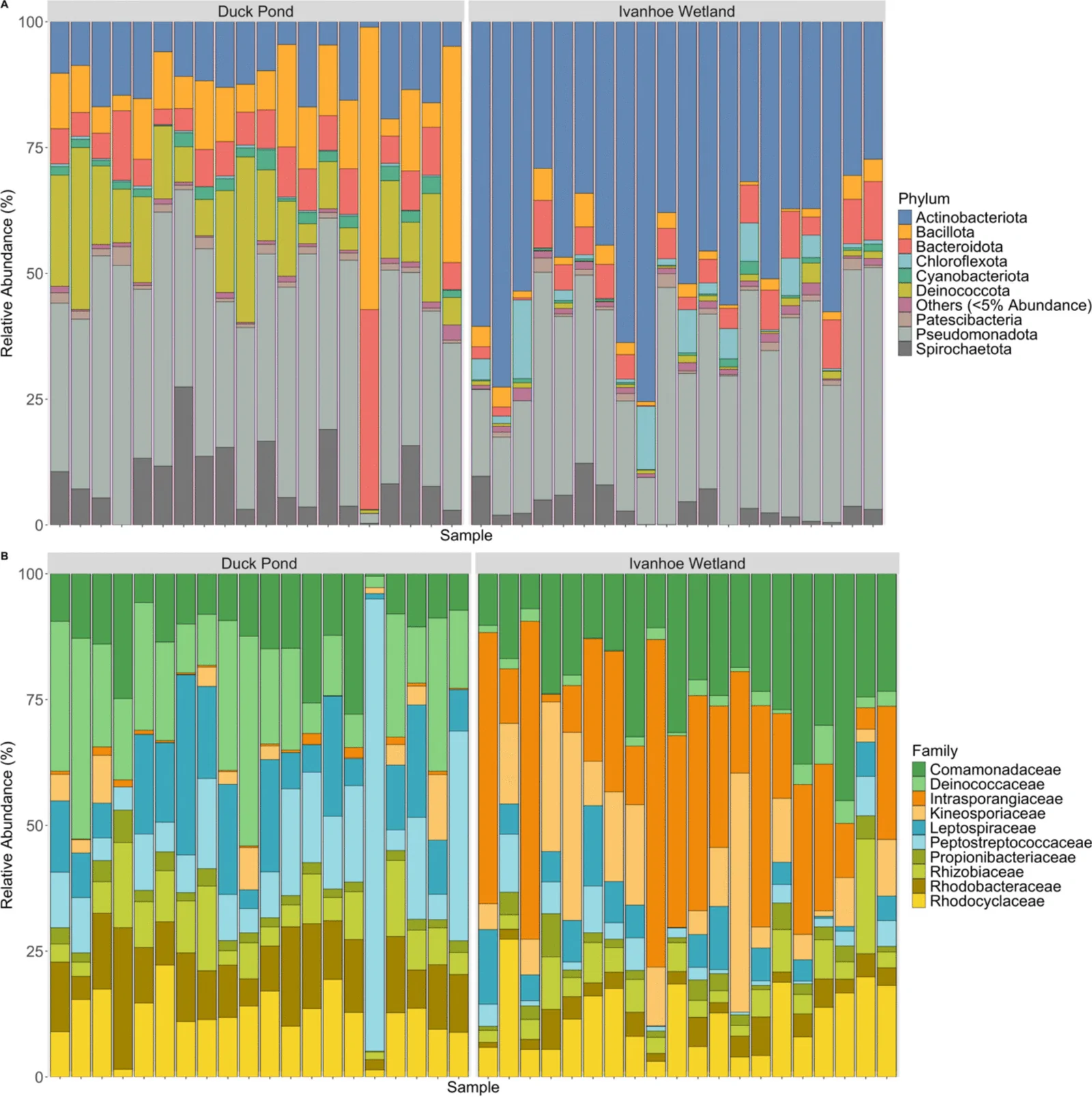
Relative abundance of the most abundant phyla (**A**), and 10 most common families (**B**) following 16 s rRNA gene sequencing of cloacal swabs from eastern longneck turtles (*Chelodina longicollis*) from the Darebin Parklands. Differences can be seen in community composition at both a Phylum and Family level between the two ponds with an increased abundance of Actinomycetota and Intrasporangiaceae in turtles from Ivanhoe Wetland

In total, 40 turtles were captured and sampled, 20 individuals from each pond. A sum of 641,363 sequences were generated after quality checking and removal of chimeras, giving an average of 16,445 sequences per sample. The taxonomic breakdown of sequence data yielded 29 bacterial phyla, 61 classes, 162 orders, 256 families, 416 genera, 339 species, and 913 ASVs. Overall, the five most predominant phyla were Pseudomonadota (35.3%), Actinomycetota (29.1%), Bacillota (8.5%), Deinococcota (7.5%), and Bacteroidota (7.25%) (Fig. 1A). However, differences in proportions of the dominant phyla existed between locations. In Duck Pond, the most dominate phyla were Pseudomonadota (38.2%), Bacillota (14%), Deinococcota (13.9%), Actinomycetota (11.3%), and Spirochaetota (9.5%), and for Ivanhoe Wetland they were Actinomycetota (47.8%), Pseudomonadota (32.3%), Bacteroidota (5.7%), Spirochaetota (3.9%), and Chloroflexiota (3.8%) (Fig. 1A). The five most abundant families across both sampling sites were Comamonadaceae (12%), Intrasporangiaceae (9.1%), Rhodocyclaeceae (8.7%), Deinococcaceae (8%), and Leptospiraceae (7.1%) (Fig. 1B). Also, like bacterial phyla, differences existed for the predominate families in turtles captured from each waterbody where in Duck Pond Deinococcaceae (14.4%), Peptostreptococcaceae (10.5%), Leptospiraceae (9.7%), Comamonadaceae (9.1%), and Rhodocyclaeceae (9.1%) dominated, and in Ivanhoe Wetland the most common families were Intrasporangiaceae (18%), Comamonadaceae (15%), Kineosporiaceae (9.4%), Rhodocyclaeceae (8.3%), and Leptospiraceae (4.3%) (Fig. 1B).

**Fig. 2:**
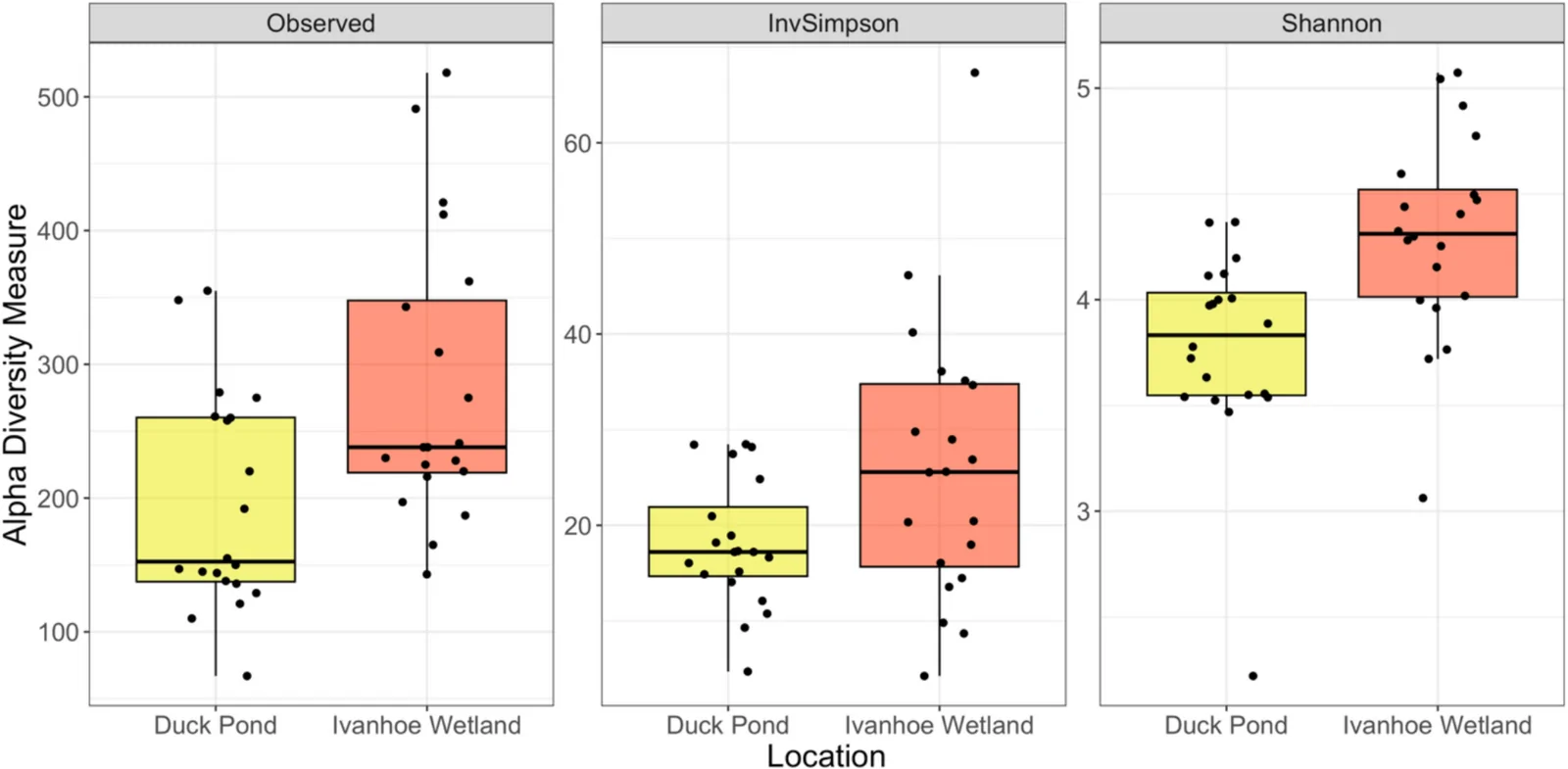
Alpha diversity metrics from cloacal swabs from eastern longneck turtles (*Chelodina longicollis*) from the Darebin Parklands. Statistically significant differences were seen between Observed (*p* = 0.01) and Shannon (*p* < 0.01), but not for Inverse Simpson (InvSimpson) (*p* = 0.01)

Analysis of alpha diversity of cloacal bacterial community composition revealed that Observed (*W* = 0.93, *p* = 0.02) and Inverse Simpson (*W* = 0.89, *p* < 0.01) were non-normally distributed while Shannon (*W* = 0.95, *p* = 0.08) normally distributed. There were significant differences in alpha diversity between localities with an increase in Observed ASVs (*W* = 105, *p* = 0.01), and Shannon diversity (*t* =  − 3.28 df = 37.95, *p* < 0.01) in turtles from IW. However, no differences were observed between locations for Inverse Simpson diversity (*W* = 132, *p* = 0.07) (Fig. 2).

**Fig. 3:**
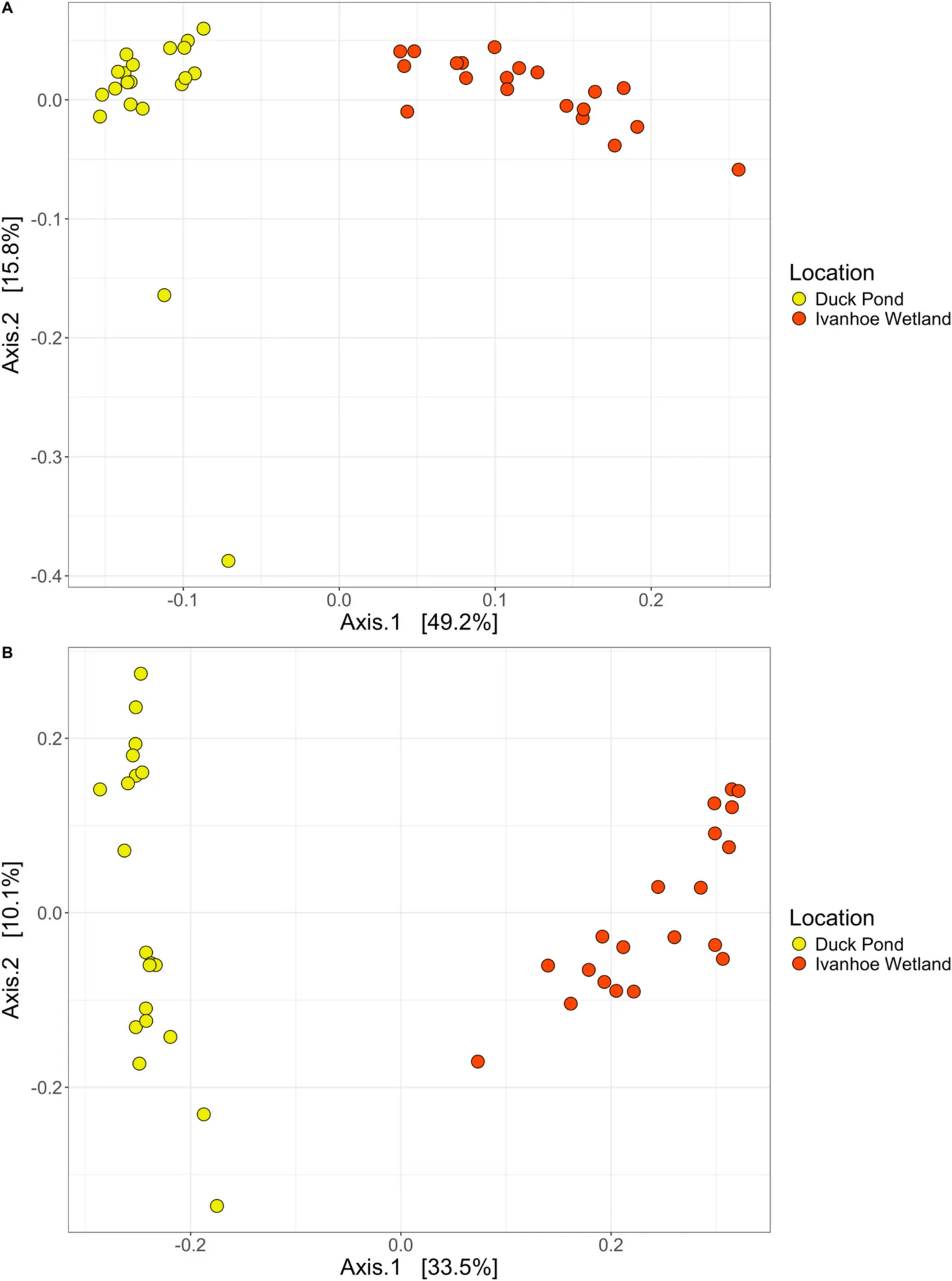
Principal coordinate analysis (PCoA) with weighted unifrac (**A**), and unweighted unifrac (**B**) distances of the ASV analysis of microbiotas from cloacal samples of eastern longneck turtles (*Chelodina longicollis*) from the Darebin Parklands. PERMANOVA revealed significant differences for both weighted unifrac (*p* < 0.01), and unweighted unifrac (*p* < 0.01)

For beta diversity, we detected significant differences in cloacal bacterial community composition between locations for both weighted unifrac (df = 1, SS_T_ = 0.57, *R*^2^ = 0.44, f.model = 29.12, *p* < 0.01), and unweighted unifrac (df = 1, SS_T_ = 2.27, *R*^2^ = 0.32, f.model = 17.53, *p* < 0.01) (Fig. 3).

### Functional Omics Results

**Fig. 4:**
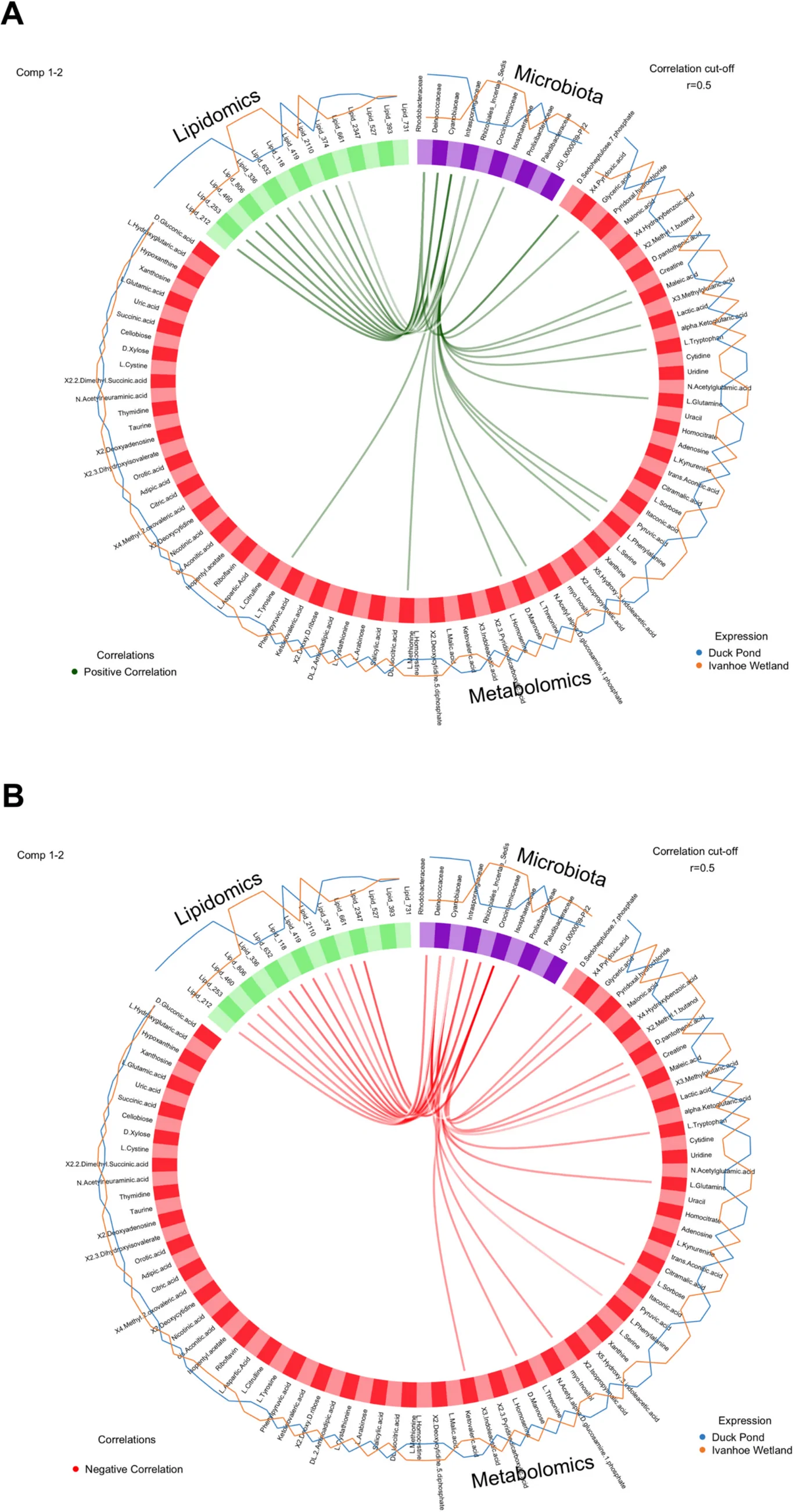
Circos plot from DIABLO performed on the study. The plot represents correlations greater than 0.5 between the variables which are represented as coloured blocks on the side of the plot; microbiota (purple), metabolomics (red), and lipidomics (green). The internal lines show positive (green) (**A**) and negative (red) (**B**) correlations while the outer lines show expression levels of each variable in each sample group

The initial dataset included 40 turtles; however, lipidomics results were not obtained for one individual originating in IW and so all data pertaining to this animal was discarded from multi-omics analysis. The initial dataset that was used for exploration in the model consisted of 130 bacterial Families, 84 targeted central carbon metabolism metabolites (metabolomics), and 1025 untargeted lipids (lipidomics). After tuning, we found the most discriminant features between the two turtle ponds included 10 microbes (5 for component 1 and 5 for component 2), 85 metabolomics (80 for component 1 and 5 for component 2), and 15 lipidomic parameters (10 for component 1 and 5 for component 2). Diagnostic diablo plots and correlation circle plots indicated a strong correlation for all datasets in our experiment with both metabolomics and lipidomics closely linked with microbiota composition (Figs. S1 and S2). Furthermore, sample plots (Fig. S3) and arrow plots (Fig. S4) indicated distinct differences existed between turtle populations for all metrics measured, microbiota, metabolomics, and lipidomics and highlighted the relationship that location, and subsequently microbiota composition, has on turtle physiology. The correlation between the variables from the three different blocks is shown in Fig. 4.

**Fig. 5:**
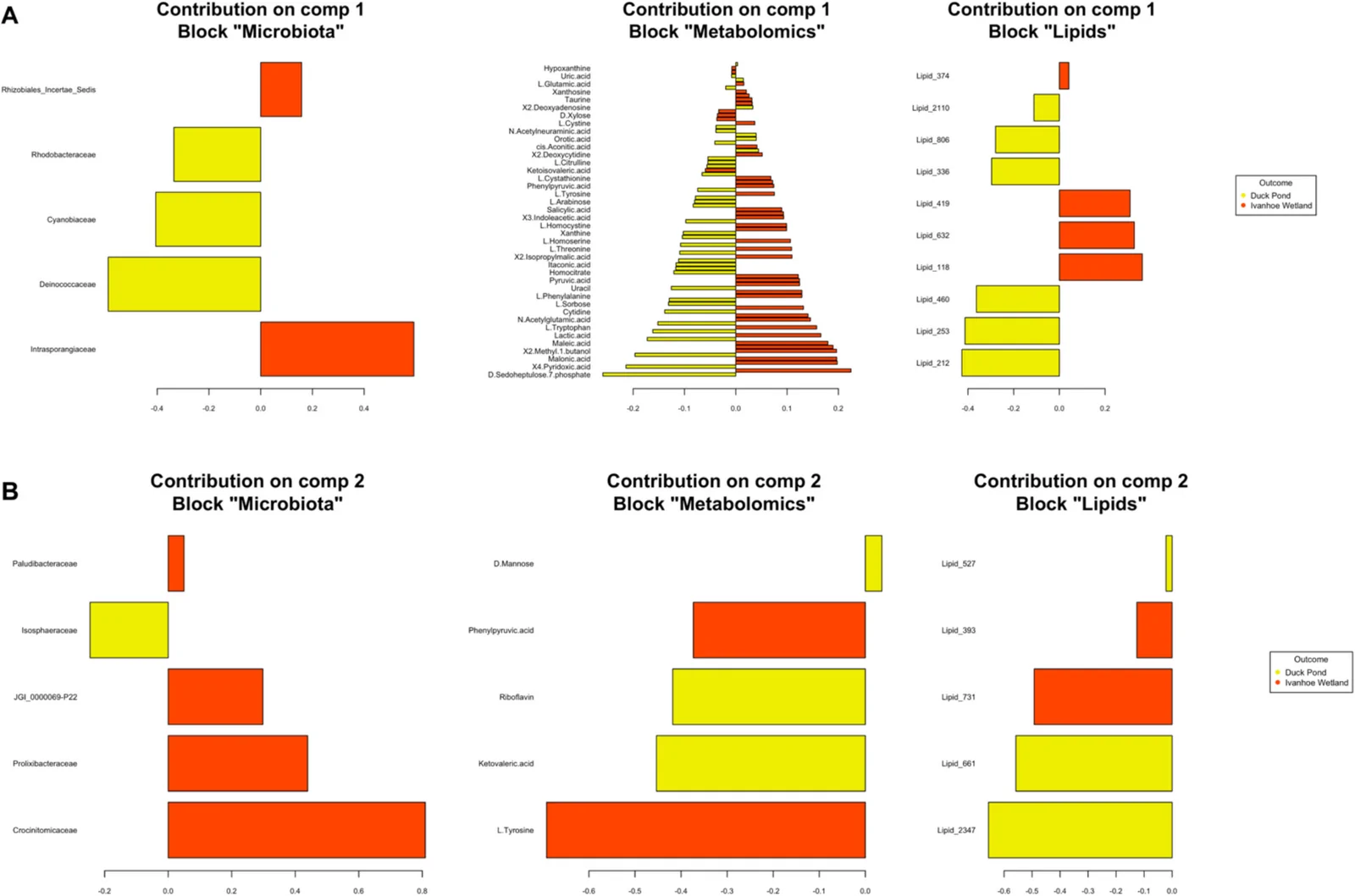
Loading plot for the variables selected by DIABLO performed on the study for component 1 (**A**) and component 2 (**B**). The most important variables are ordered from bottom to top. The length of the bar on each plot represents the absolute value of the loading weight for that variable, indicating how much it contributes to the component being analysed

We discovered that six bacterial families were positively correlated with 11 lipids and 14 metabolomics (Fig. 4A), and that seven bacterial families had a negative correlation with 12 lipids and 13 metabolomic compounds (Fig. 4B). The appearance of the loading plots indicates that component 1 does not particularly discriminate between sites with an even spread between each of the blocks and the most important families responsible for differences between sites included Intrasporangiaceae, Deinococcaceae, Cyanobiaceae, Rhodobacteraceae, and Rhizobiales incertae sedis (Fig. 5A). For component 2, most discriminatory bacterial families originated from IW, and included Crocinitomicaceae, Prolixibacteraceae, JGI_0000069-P22, Isosphaeraceae, and Paludibacteraceae (Fig. 5B). In this component, lipids from animals originating in DP were the most discriminatory while there was an even spread of metabolomics between the two sites (Fig. 5B).

**Fig. 6:**
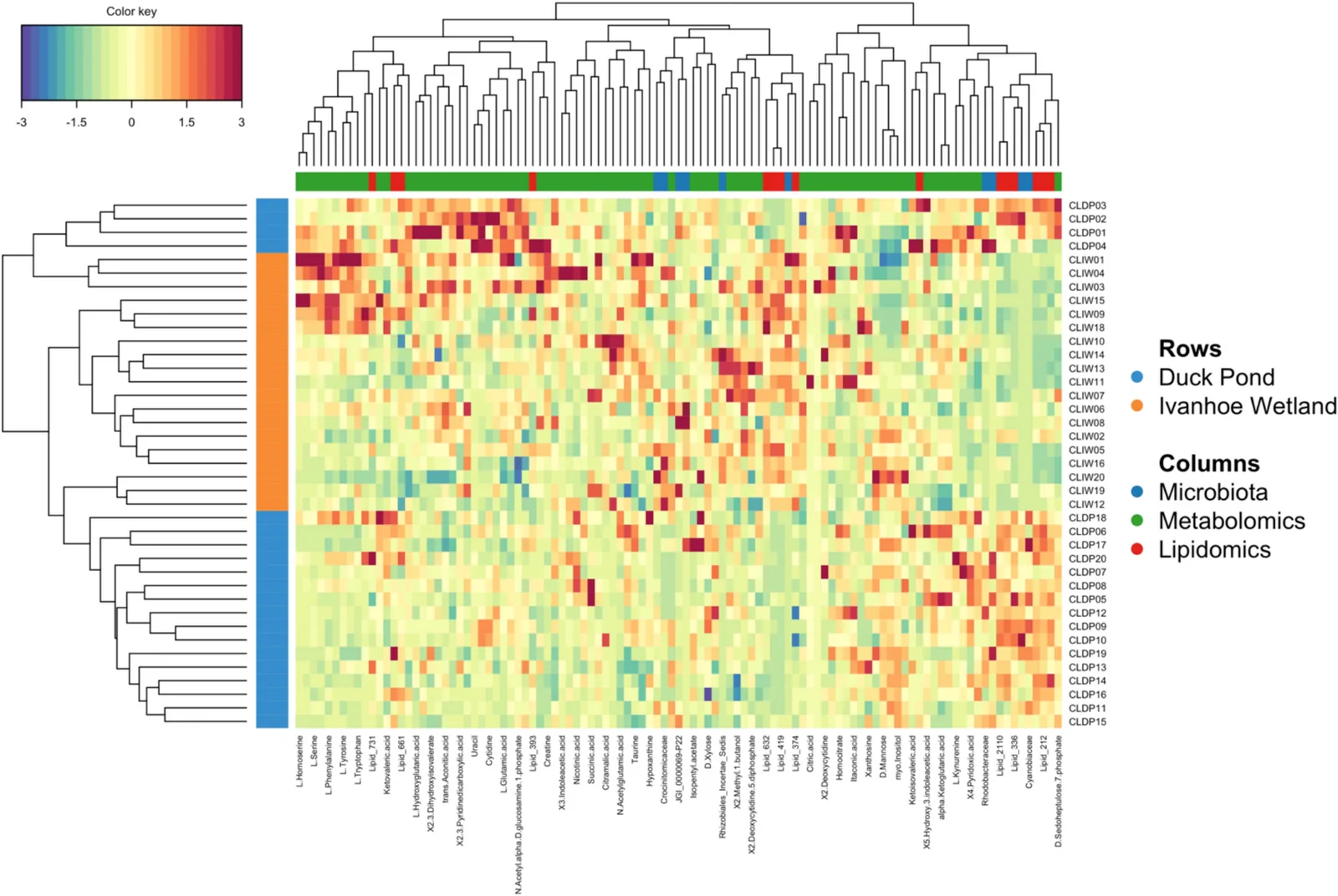
Clustered image map (CIM) for the variables selected by DIABLO performed on the study. The CIM represents samples in rows and selected features in columns

The unique multi-omic signature of the turtles was depicted using a CIM (Fig. 6). Hierarchical clustering of the samples almost perfectly classified turtles into their locations except for six animals (three from DP and three from IW), which were closely aligned. Most correlations were strongly positive, with only a handful of predictors having a strong negative correlation.

To assess the performance of the proposed omics profile, we calculated an overall error rate of 0.01 for component 1, which improved to < 0.01 with the addition of a second component, and an overall majority vote of 0.01 for component 1, which improved to < 0.01 with the addition of component 2 to the model. Additionally, the receiver operating characteristic (ROC) curve analysis showed that the optimal omics profile for microbiota block was 1 (*p* < 0.01), metabolomics was 1 (*p* < 0.01), and lipidomics was 0.99 (*p* < 0.01) (Fig S5c). These results support the above-selected features as representative omics profiles of the turtles from their respective ponds in this investigation.

**Fig. 7:**
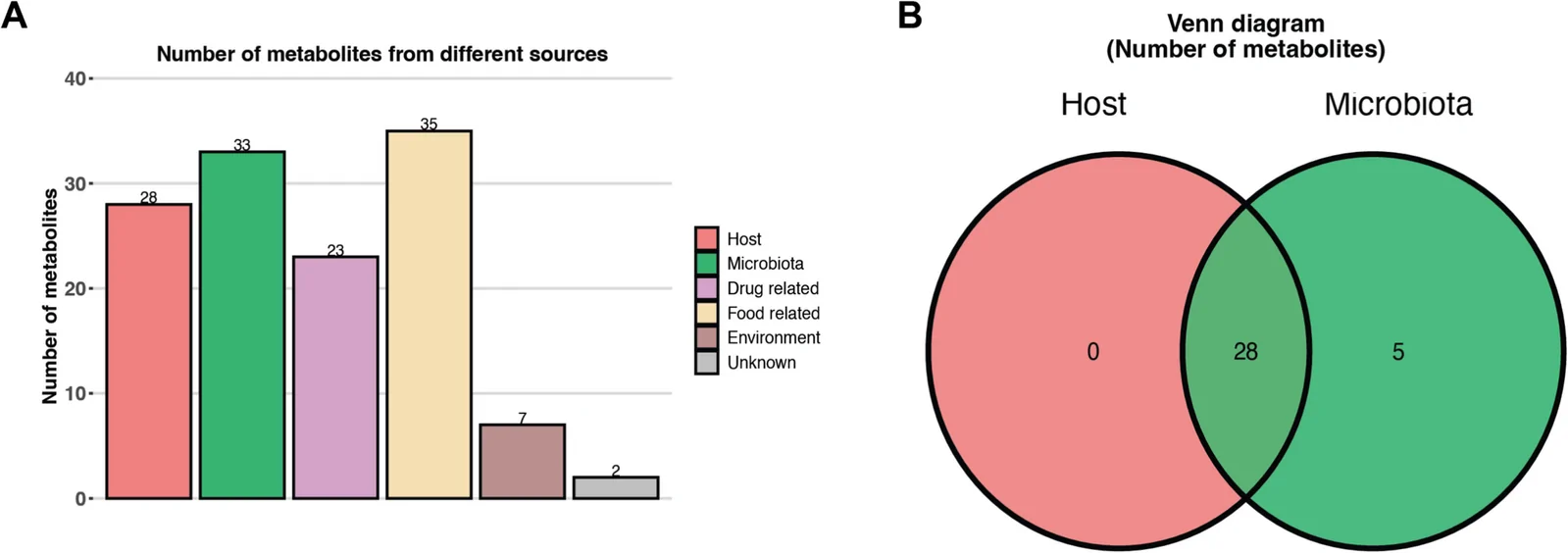
Number of identified metabolites from different sources as determined by MetOrigin performed against the red-eared slider (*Trachemys scripta elegans*) genome. Breakdown of possible origins of metabolomic products (**A**), and Venn diagram indicating possible origin of 33 metabolites attributed to either host or microbiota (**B**)

We were able to locate KEGG or Human Metabolomic Database (HMDB) identification numbers for 80 of our discriminative metabolites. The metabolite host-microbiota origin analysis indicated that of our significant metabolites, 28 may have originated from the host, and 33 may be microbiota-based (Fig. 7A). Interestingly, none of the identified metabolites were considered host only, with 5 of definite microbiota origin and 28 possibly arising from either host or microbiota (Fig. 7B) (Table S2).

## Discussion

To our knowledge, this is the first investigation to explore the relationship between cloacal microbiota and serum metabolome in a wild reptile. We were able to show that location has a significant effect on microbial composition and subsequently the metabolic profiles of Australian freshwater chelonians. In our study populations, we found a predominance of the bacterial phyla Pseudomonadota, Actinomycetota, and Bacillota and of the families Comamonadaceae, Intrasporangiaceae, and Rhodocyclaeceae (Fig. 1). In our investigation, we found that significant differences existed between DP and IW for both alpha and beta diversity and that ten bacterial families were primarily responsible for driving these differences with the most important of these being Intrasporangiaceae, Deinococcaceae, Cyanobiaceae, Crocinitomicaceae, Prolixibacteraceae, and Isophaeraceae (Fig. 5A, B). Discovery of such disparate microbiotas in animals from these ponds was an unexpected finding given the proximity of the waterbodies to each other. This is further complicated by the fact that eastern longneck turtles are capable of long terrestrial migrations [55], and so it is possible that there is movement of animals between sites; however, this has never been confirmed. No attempt was made to investigate factors responsible for observed variation, as this investigation was a small component of a larger project involving multiple freshwater turtle species and we did not anticipate that we would encounter such disagreement between these animals sourced from nearby locations.

Factors that may be driving the observed variance in microbial populations are multifactorial, but one possible explanation may include a difference in environmental pollutants between the ponds. Macquarie River turtles (*Emydura macquarii macquarii*) exposed to elevated per- and polyfluoroalkyl substance (PFAS) levels have shown disruption of their gut microbiotas with impacted individuals having an increase in the Bacillota-Bacteroidota ratio from 1.4 to 5.5 with a correlated increase in stress and inflammatory-related metabolites [56]. Similarly, exposure to high concentrations of organophosphorus flame retardants (OPFRs) such as triphenyl phosphate (TPhP) has been shown to alter the proportions of specific intestinal bacterial genera and affect hepatic antioxidant and metabolomic abilities in Chinese soft-shelled turtles (*Pelodiscus sinensis*) [57]. Additionally, perturbations in the gut microbiota composition of Chinese soft-shelled turtles have been experimentally induced when both eggs and hatchling turtles were exposed to nanoplastics, with affected individuals having a relative increase in the proportions of Pseudomonadota, Bacteriodota, and Fusobacteriota with a concurrent decrease in the relative abundance of Bacillota [58]. We did not see similar patterns of proportional changes in microbial populations between our turtle ponds, nor did we detect any evidence of impaired hepatic function [59]; however, this does not mean that we can rule out differences in environmental contamination as a cause of these dissimilarities. Future investigations at this site should attempt to elucidate features that may be contributing to these differences, and to determine that if turtles migrate between ponds, whether their microbiotas assimilate to reflect those of conspecific resident turtles.

The phyla Pseudomonadota, Bacillota, and Bacteroidota are regularly reported to dominate the cloacal microbiota of freshwater turtles in varying proportions including Blanding’s turtles (*Emydoidea blandingii*) [27], painted turtles (*Chrysemys picta*) [28], Macquarie River turtles [33], Chinese three-keeled pond turtles (*Mauremys reevesii*) [29], false map turtles (*Graptemys pseudogeographica*) [30], western pond turtles (*Emys marmorata*) [31], big-headed turtles (*Platysternon megacephalum*), and Beale’s eyed turtle (*Sacalia bealei*) [32], as well as in all species of marine turtles [7], indicating that there might be some consistency in microbial communities across chelonian species at a high taxonomic level. An unusual finding in our investigation was the relatively high proportion of Actinomycetota (47.2%) in animals captured from IW (Fig. 1A). Although Actinomycetota are a common component of the vertebrate microbiota [60], they are typically present in lower proportions than was found in IW turtles. Members of the Actinomycetota are important pathogens of humans and animals [60], and in humans an increase in the proportion of Actinomycetota in the gut microbiota has been linked to intestinal disorders such as Crohn’s disease and ulcerative colitis [61]. However, this is not consistent across all investigations involving humans, and for some cohorts, an increase in relative abundance of Actinomycetota is associated with differences in diet, habits, and clinical history [62]. There were no obvious indications that turtles captured from IW were clinically unwell, and it remains to be seen what their unique cloacal microbiota composition means for their gastrointestinal health and what the driving forces behind this observed variance was.

Differences in Observed ASVs and Shannon diversity metrics were discovered between our study populations with turtles from IW having a larger number of Observed ASVs as well as increased bacterial diversity (Fig. 2). However, interpreting the physiological consequences for these differences when examined in isolation are difficult as there are conflicting views on the significance that alpha diversity metrics play in determining health and fitness in vertebrates. While low diversity has been correlated with disease in humans and animals [63, 64], there is some evidence that high diversity can result in microbial antagonism which subverts microbiota harmony and decreases productivity [65]. Furthermore, in some species with highly specialised diets, such as the giant panda (*Ailuropoda melanoleuca*), high diversity is associated with nutritional distress and gastrointestinal upset [66]. In the western pond turtle (*Actinemys marmorata*), a decrease in habitat quality was associated with a decrease in cloacal alpha diversity but no attempts were made to associate these changes with turtle health or physiology [31]. Therefore, to untangle the complex role that microbial diversity plays in determining host physiology, a more holistic approach needs to be adopted where investigators probe beyond simple compositional data and explore functional microbial testing. In our analysis, we were able to demonstrate a clear link between microbiota diversity and serum metabolome indicating that specific combinations of bacteria have a significant effect on host physiology (Fig. 4). However, what we were unable to determine is the enduring effects that these differences have on host fitness and health. This is because our samples were obtained at a single point in time and may not be representative of the host-symbiont relationship over the entire span of an individual turtle’s life. Despite these limitations, these findings are an important first step in understanding how microbes exert their influence on chelonians, and future investigations should attempt to further explore these interactions by using longitudinal studies or by manipulation of the microbiota through antibiotic administration or faecal transplants [65].

Two of the most significant bacterial families in our investigation were Intrasporangiaceae, which was both positively and negatively linked to the production of metabolomics, and Deinococcaceae which was positively and negatively associated with lipid production in our turtles (Fig. 4A, B). In humans, the role that these families play in maintaining gut health is poorly defined but Intrasporangiaceae has been identified as a common opportunistic pathogen in patients suffering from bacteraemia as a consequence of decompensated liver cirrhosis [67], and members of the family Deinococcaceae are being investigated for their potential protective qualities against oxidative damage to keratinocytes [68]. In this investigation, Intrasporangiaceae was diminished in turtles from DP, while conversely, Deinococcaceae was increased in these animals, which may possibly be linked to differences in prey availability in this pond. Interestingly, a decrease in relative abundance of Intrasporangiaceae has been linked to the development of Parkinson’s disease in humans [69], but we did not observe any evidence of neurodegenerative disorders in any of our turtles or any other obvious pathologies. Further study is required to understand the interplay between Intrasporangiaceae and Deinococcaceae and their role in influencing chelonian health.

We were able to show that several of the most significant identified metabolites were potentially of microbiota origin (Table S2). However, these results must be interpreted with a high degree of caution as our reference species was the red-eared slider, which is a cryptodiran chelonian, and with the exception of the pig-nosed turtle (*Carettochelys insculpta*), all Australian freshwater chelonians are pleurodirans [70]. The origin of the clades Pleurodira and Cryptodira occurred in the early Jurassic, nearly 200 million years ago, and Australian pleurodiran turtles have been further separated for approximately 110 million years from all other species [71]. Given the strong links between phylogeny and microbiota [7–10], it is plausible that the unique biotic and abiotic factors that have moulded Australian turtle biodiversity have resulted in fundamental differences in metabolic pathways in eastern longneck turtles in comparison to the available reference species. Additionally, all of the reference species in MetOrigin were either herbivorous or omnivorous, and the eastern longneck turtle is an obligate carnivore [55], which may also affect this analysis. Further work on metabolites and their origins in Australian freshwater chelonians is warranted to explore these principles in greater detail.

Ascribing functional significance to differences in metabolite concentrations in our investigation is difficult because there have been limited investigations into their specific role in physiological processes in freshwater chelonians [56, 72]. However, we were able to show that tryptophan and pyruvic acid were positively associated with turtles from IW (Fig. 4A). In vertebrates, these metabolites are important components of glycine, serine, and threonine metabolism which are utilised for various functions within the body, primarily involving the liver and kidneys [73]. Similarly, we found that some metabolites associated with pyruvate metabolism (important for energy production in vertebrates [74]), tryptophan metabolism (important for modulating immunologic tolerance [75]), and glycolysis (responsible for the anaerobic production of ATP [76]) were also positively expressed in IW turtles (Fig. 4A). Whether or not these differences represent actual increases in physiological performance in animals from IW in comparison to DP turtles remains unknown. Further research into the biochemical pathways of cellular metabolism in pleurodirans is required to fully appreciate the functional consequences of these correlations.

A limitation of this investigation was the use of cloacal swabs for collection of the microbiota data which may have some effect on the robustness of our findings. Faecal samples may not accurately represent the microbial composition of the entire gut due to variations in microbial distribution along the gastrointestinal tract, particularly in regions such as the small intestine [77]. Swabs, while useful for targeted sampling, may only capture a fraction of the microbial diversity present in an area, potentially leading to an underrepresentation of certain taxa [78]. Additionally, these sampling techniques often lack the temporal resolution needed to observe dynamic changes in microbial communities over time, limiting their ability to elucidate causal relationships [79]. Addressing these limitations is essential for improving the reliability and applicability of microbiome research; however, in many instances, there are no realistic alternatives and as such data must be assessed with these caveats in mind. In Australia, all native freshwater turtles are protected species and therefore the only option available to obtain samples from live individuals is via cloacal swab.

In conclusion, this study highlights the intricate interplay between microbes and metabolomics, revealing how microbial communities influence host metabolism and physiology. The integration of our data shows that the microbiota of turtles can be metabolically and compositionally distinct depending on their point of origin. By using a multi-omics approach, we have identified key microbial taxa and correlated metabolic products that may serve as potential biomarkers for various physiological states. Our study is the first investigation to highlight these clear links in a wild reptile and gives further insight into chelonian physiology. Future research should continue to explore these relationships with an emphasis on longitudinal and manipulative studies to further elucidate the dynamic host-microbiota relationship, particularly in understudied taxa such as chelonians.

## Supplementary Information

Below is the link to the electronic supplementary material.

Supplementary file1 (DOCX 29340 KB)

Supplementary file2 (XLSX 82 KB)

Supplementary file3 (CSV 16 KB)

## Data Availability

All data presented here and in the supplementary material have been submitted to The National Center for Biotechnology Information (www.ncbi.nlm.nih.gov) and can be accessed on request from the authors.
