# Supplementary material for: Pondering Ponds: Exploring Correlations Between Cloacal Microbiota and Blood Metabolome in Freshwater Turtles
Source: Microb Ecol. 2025 May 23;88(1):50. doi: 10.1007/s00248-025-02556-7 (PMC12098208; doi:10.1007/s00248-025-02556-7)
Supplement: Supplementary file 1 — Supplementary file1 (DOCX 29340 KB) [file 248_2025_2556_MOESM1_ESM.docx]

**
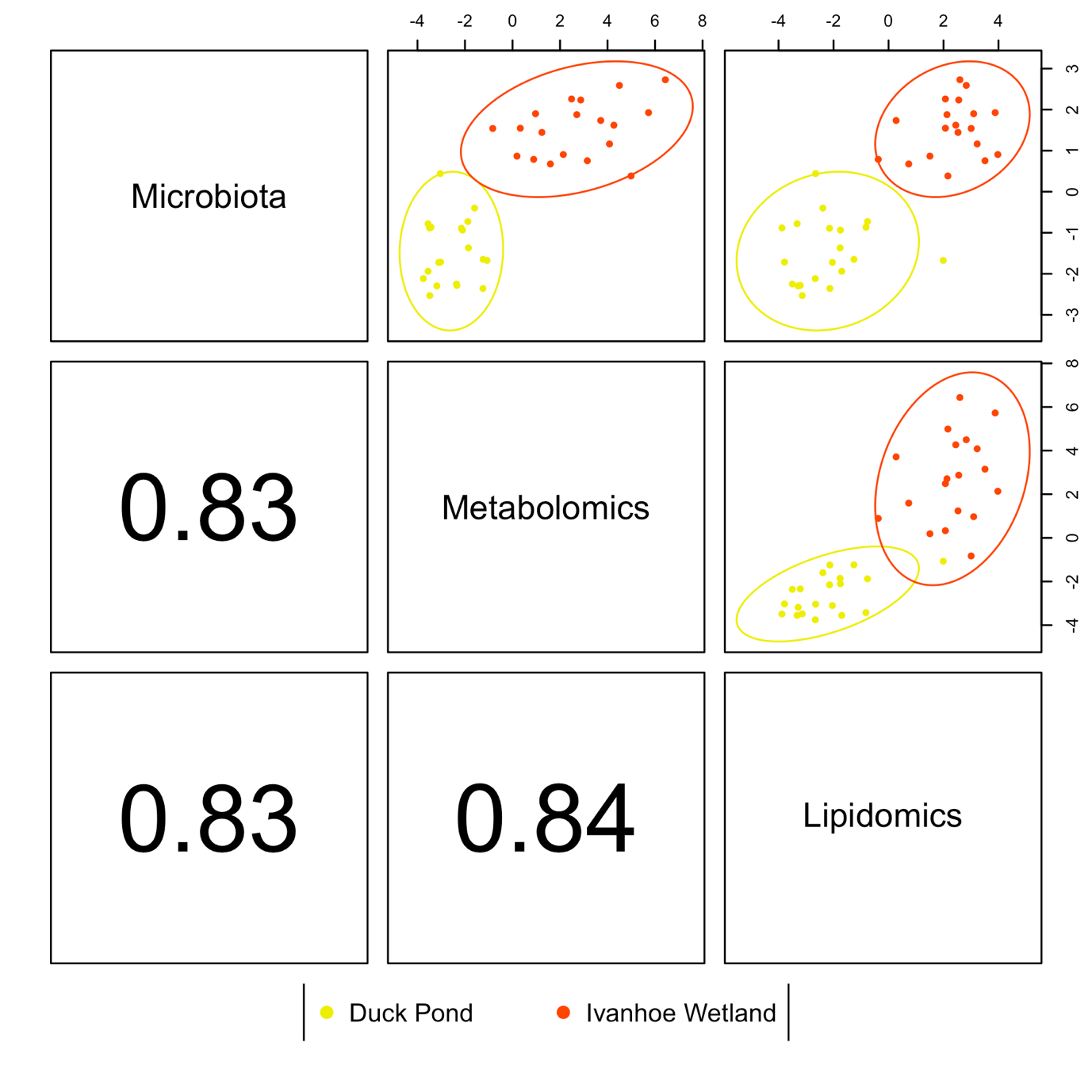
**

**Figure S1.** Diagnostic plot from multiblock SPLSDA applied to the data. Samples are represented by component 1 for each data set (Microbiota, Metabolomics and Lipidomics). Samples are coloured by location (Duck Pond and Ivanhoe Wetland). The bottom left numbers indicate the correlation coefficients between each dataset.


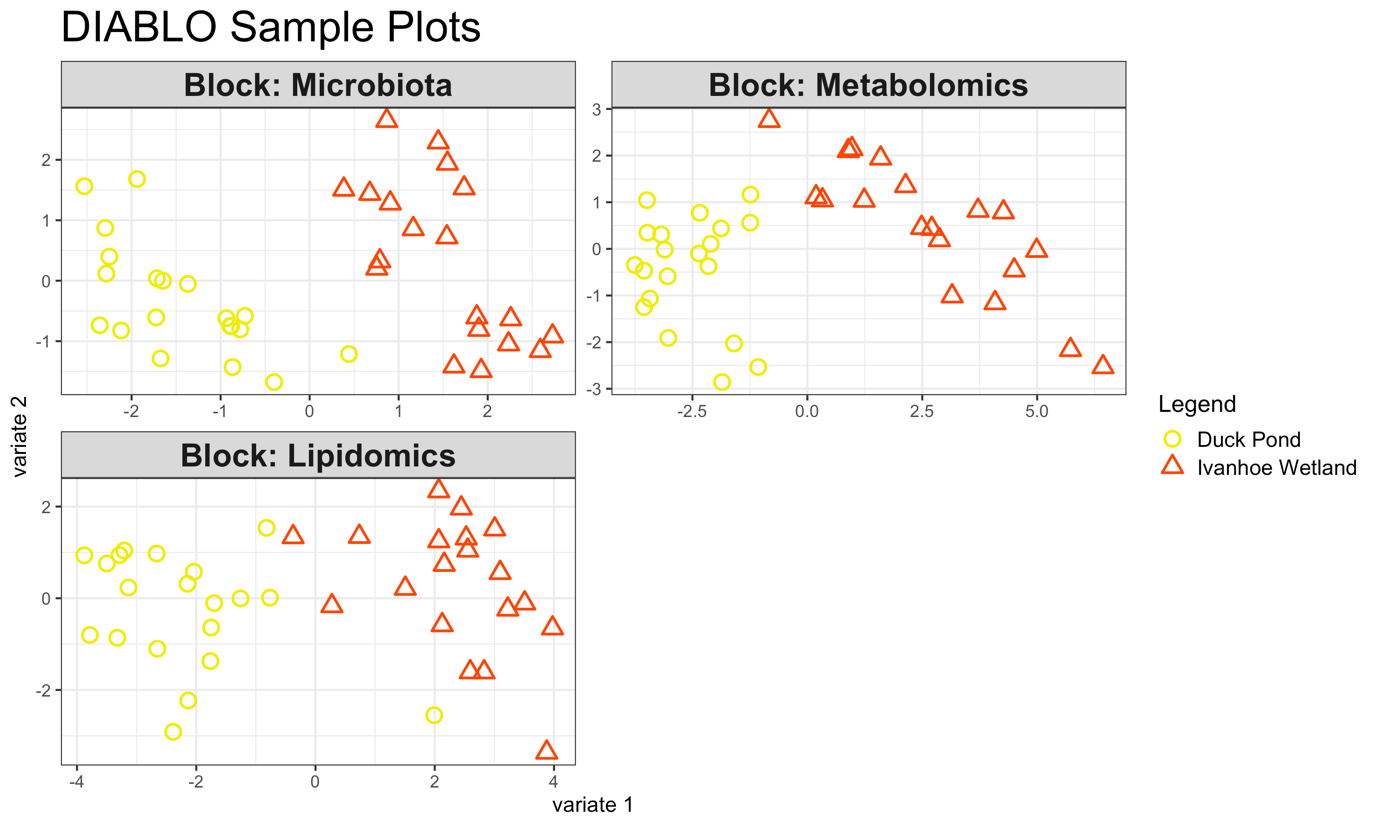


**Figure S2.** Sample plot from multiblock SPLSDA performed on the data. The samples are plotted according to their scores on the first 2 components. Samples are coloured by location (Duck Pond and Ivanhoe Wetland) and it shows the degree of agreement between the different datasets and the discriminative ability of each.


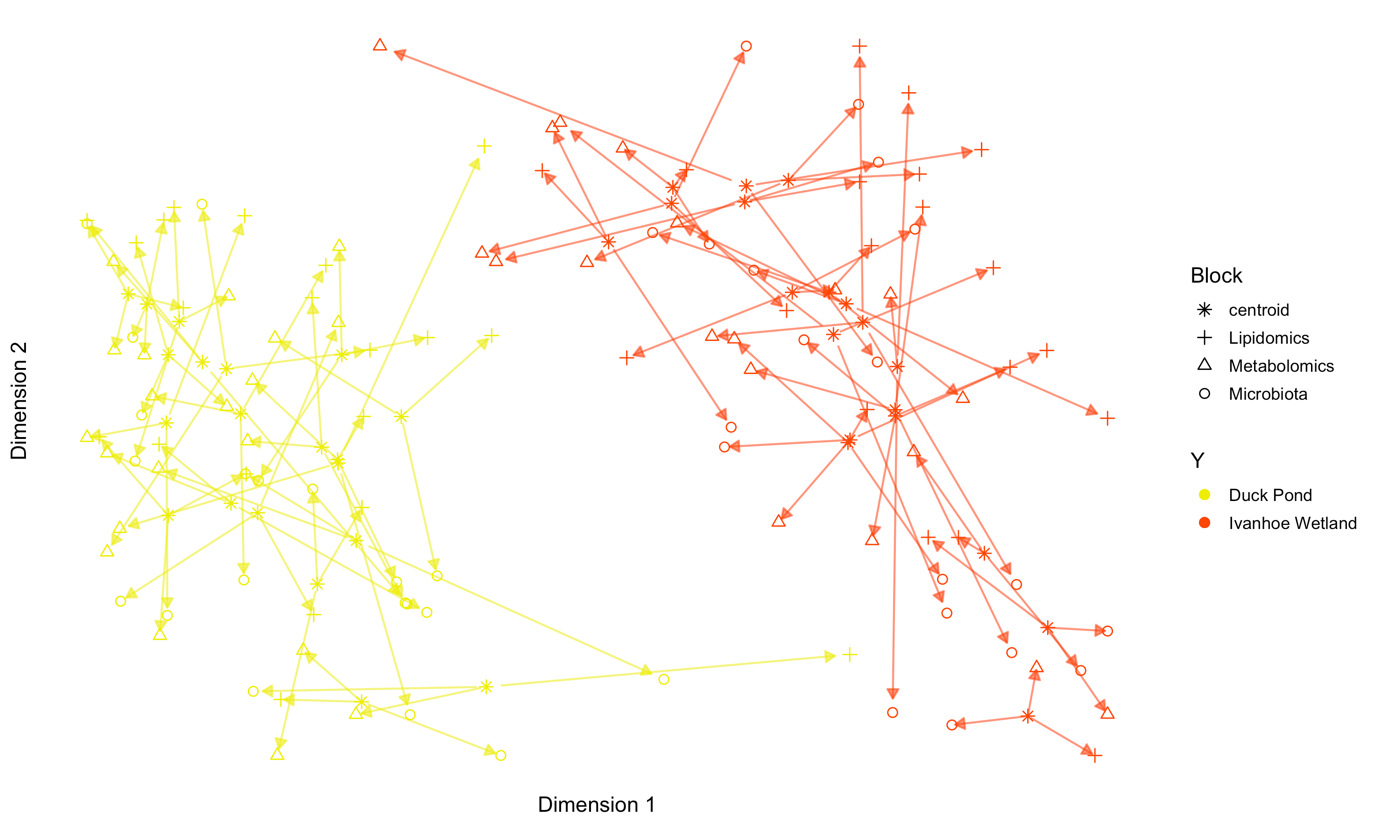


**Figure S3.** Arrow plot from multiblock SPLSDA performed on the data. Arrows further from their centroid indicate some disagreement between the datasets. Samples are coloured by location (Duck Pond and Ivanhoe Wetland).


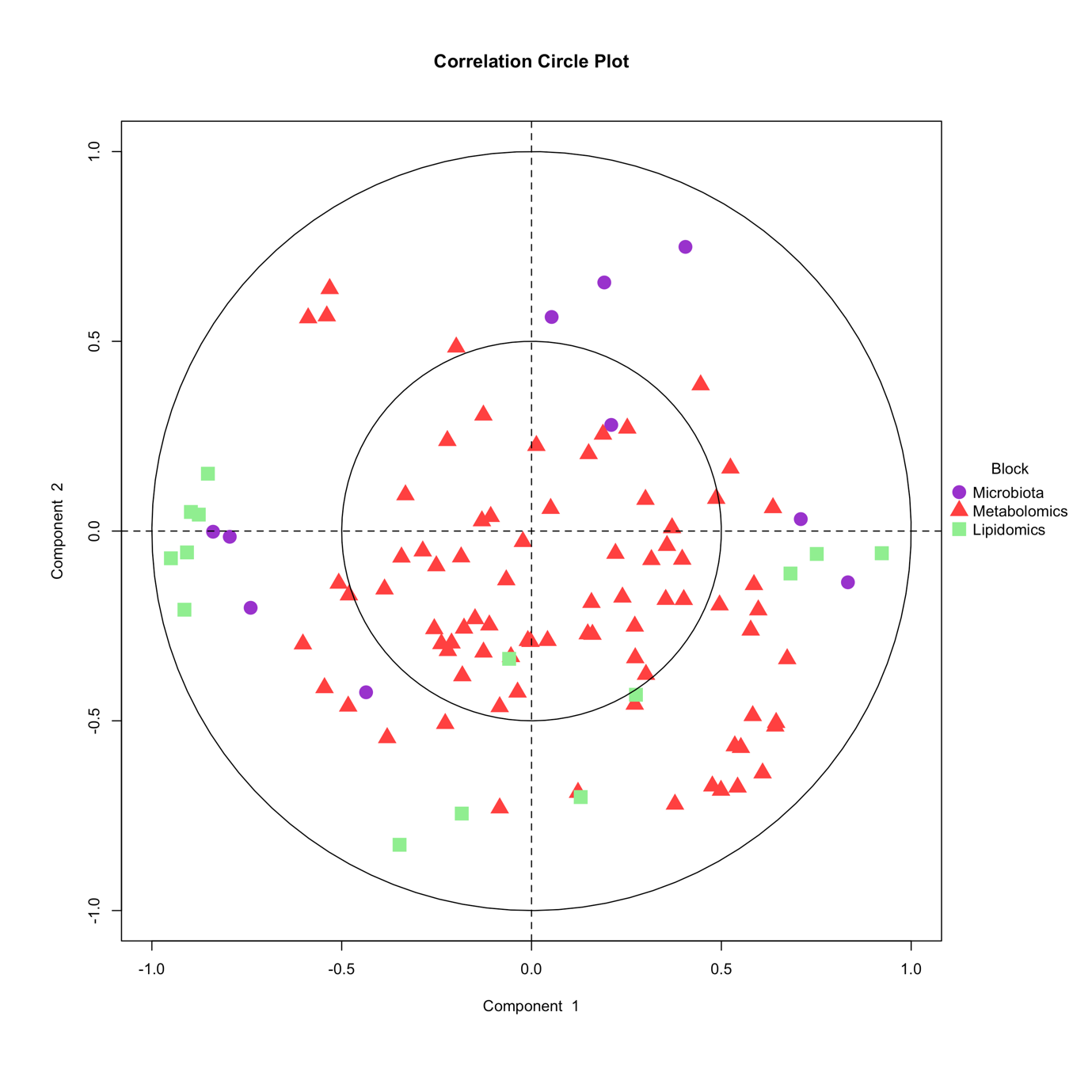


**Figure S4.** Correlation circle plot from multiblock SPLSDA performed on the data. Clusters of points indicate a strong correlation between variables.


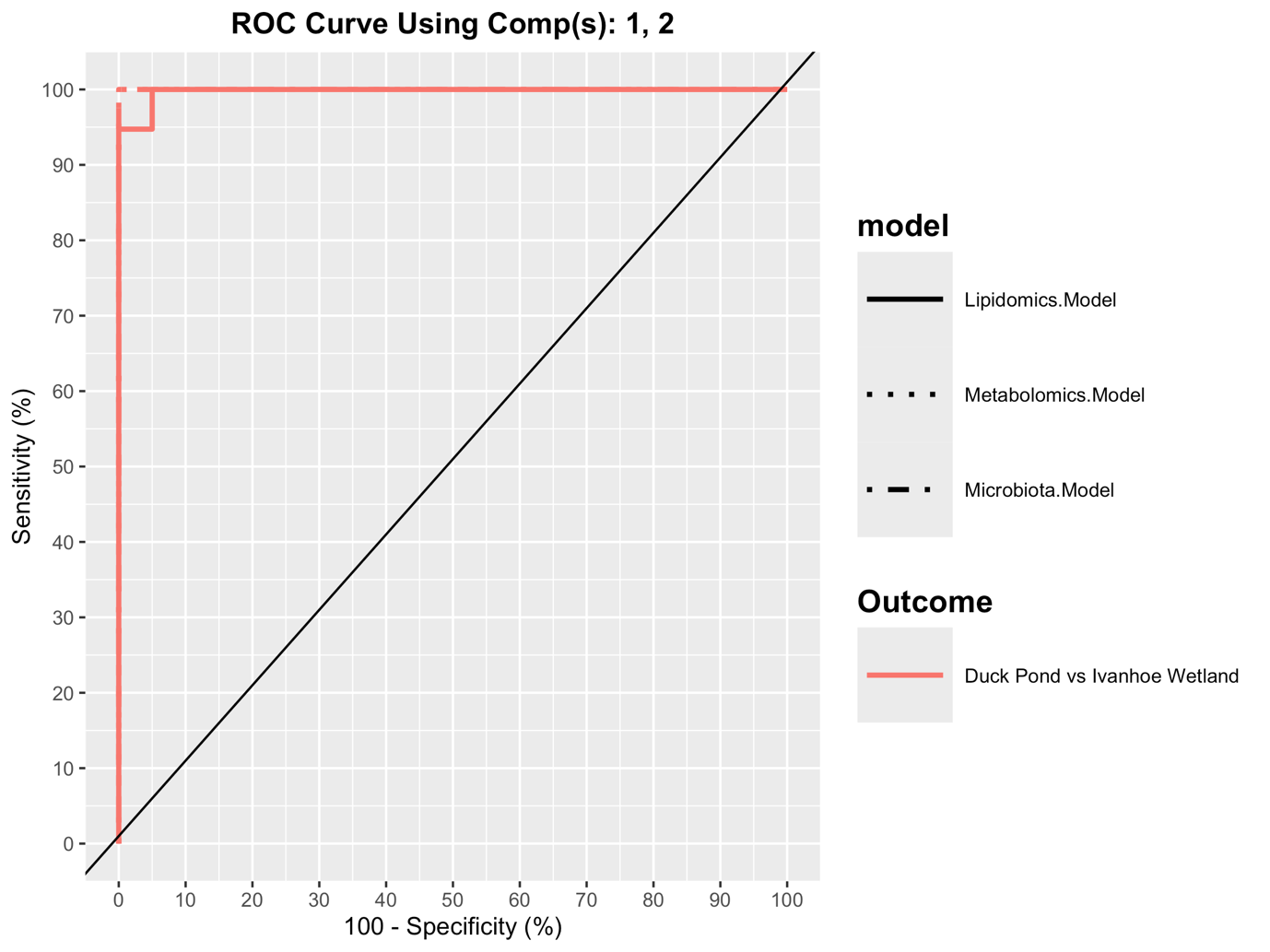


**Figure S5.** Receiver operating characteristic (ROC) and area under the curve (AUC) based on multiblock SPLSDA performed on the data for microbiota, metabolomics, and lipidomics.
